# Highly elastic and flexible transparent conductive films derived from latex copolymerization: P(SSNa-BA-St)/PEDOT/graphene†

**DOI:** 10.1039/c9ra09099a

**Published:** 2019-12-20

**Authors:** Bo Huang, Xinxin Luo, Qichao Zou, Suxiao Wang, Jinzhi Zhang

**Affiliations:** Ministry of Education Key Laboratory for the Synthesis and Application of Organic Functional Molecules, Hubei Collaborative Innovation Centre for Advanced Organic Chemical Materials, College of Chemistry and Chemical Engineering, Hubei University Wuhan 430062 China wsx@hubu.edu.cn zjz4000@126.com

## Abstract

We reported an innovative transparent, elastic and flexible conductive composite materials P(SSNa-BA-St)/PEDOT/graphene which were prepared by using P(SSNa-BA-St) latex as template for PEDOT polymerization and graphene doping. This P(SSNa-BA-St)/PEDOT/graphene film exhibited highly transparent, good water resistance, low moisture adsorption, highly elastic and highly conductive properties, which can serve as a practical approach to fabricate the flexible, conductive and transparent films for wearable and implantable electronic devices, and photovoltaic cells.

## Abstract 2

We reported an transparent, water resistant, and flexible conductive materials P(SSNa-BA-St)/PEDOT/graphene and their conductivity are due to the large surface area to polymerize the extended PEDOT chain on the nanoparticles.

## Graphical abstract

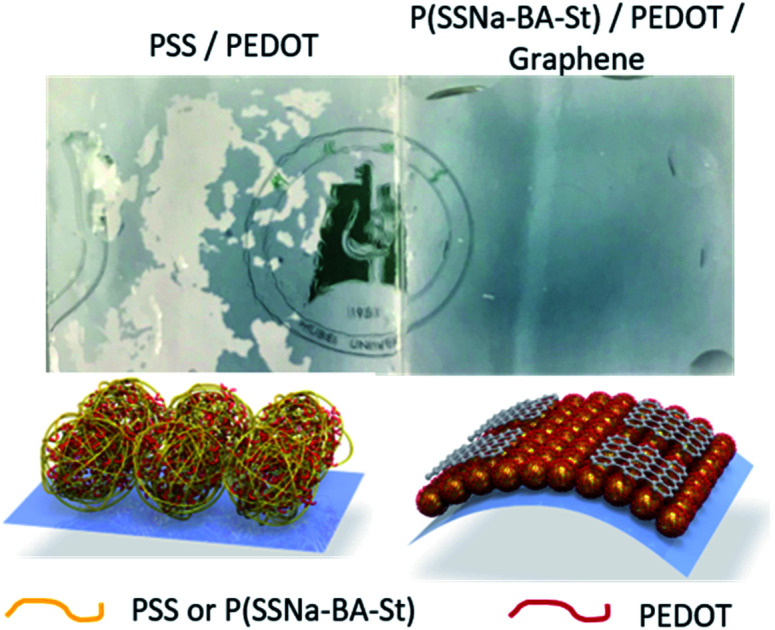

## 1. Introduction

The polyelectrolyte complex poly(3,4-ethylenedioxythiophene) (PEDOT)/poly(styrene sulfonate) (PSS) is one of the most well-known organic conductors with highly conductive, largely transmissive to light, processible in water and flexible.^1^ It has been used for electrostatic coatings, light emitting diodes, supercapacitors, and flexible photovoltaic cells, *etc.*^2–4^ To improve the solubility of semiconductor PEDOT, PSS has been used to stabilize PEDOT as a counterion and charge compensator which provide a matrix for PEDOT to form an aqueous dispersion. Basically, EDOT can be chemically polymerized in a PSS solution to form PSS/PEDOT dispersion.^5^ However, PSS as an insulator could strongly decrease the conductivity of the PSS/PEDOT.^6^ Therefore, increasingly intensive work is going on to enhance the conductivity of PSS/PEDOT by organic compounds, salts, acid, zwitterions and anionic surfactant, *etc.*^7–9^ Secondary doping by polar solvents is also used to increase the conductivity, which is attributed to morphological changes. Doping the polar solvents could trigger phase separation between the conducting PEDOT chains and insulating PSS chains, leading to an interconnected network of elongated PEDOT grains and removal of insulating PSS chains for electrical conductivity enhancement.^10–12^

Graphene has also been used as the candidates for making conductive composite with PEDOT/PSS.^13^ Directing mixing the water soluble graphene oxide (GO) with PEDOT/PSS was limited by the poor conductivity of GO,^14^ therefore, the water insoluble reduced graphene oxide (RGO) was carefully *in situ* polymerized with PEDOT/PSS to make the highly conductive PEDOT/PSS/graphene composite.^15^

On the other hand, much work has been devoted to increasing the flexibility and stretchability of PEDOT/PSS which is a characteristic required for a range of applications such as wearable, implantable and large-area electronic devices.^16^ Blending with polymers is one of the most efficient route to increase the flexibility of PSS/PEDOT. Poly(ethylene glycol) (PEG), poly(ethylene oxide) (PEO), poly(vinyl alcohol) (PVA), and polyurethane (PUR) have all been used.^16–19^ Chiu *et al.* designed highly flexible PEDOT:PSS–P(styrene-*co*-butyl acrylate) (P(St-BA)) dispersions by using PEDOT:PSS as stabilizer for P(St-BA) latex, but the P(St-BA) latex were micro-sized range with polydispersity due to the weak stabilizing capability of PEDOT:PSS.^20,21^ Chiu *et al.* also designed PEDOT:PSS–(P(St-BA)) dispersions by applying dodecylbenzene sulfonic acid (DBSA) as surfactant.^22^ However, one disadvantage of these polymer blended film is the decrease in conductivity with increasing amounts of insulating polymer. And the blending approach is even more difficult with hydrophobic elastomers.

Herein, P(SSNa-BA-St) latex were used as template for both graphene and PEDOT to prepare the transparent, highly elastic and conductive P(SSNa-BA-St)/PEDOT dispersions. SSNa served as both stabilizer and monomer for P(SSNa-BA-St) copolymerization latex, therefore, no extra surfactant is needed and monodispersed nanoparticles are formed. The graphene locating on the surface of P(SSNa-BA-St)/PEDOT particles through the hydrogen bonding and stacking interaction between not only PSS and graphene but also PSt and graphene. P(SSNa-BA-St) latex with approximately 128 nm sizes have large surface area to polymerize the extended PEDOT chain on the surface of the nanoparticles, which can provide much higher conductivity than PSS/PEDOT that having grains with hydrophobic coiled PEDOT chains in the core and hydrophilic insulating PSS as the shell. Moreover, the P(SSNa-BA-St)/PEDOT/graphene presented here exhibited transparent, good water resistance, low moisture adsorption and highly elastic properties, which can serve as a practical approach to fabricate the flexible, conductive and transparent films for wearable and implantable electronic devices, and photovoltaic cells.

## 2. Experimental

### 2.1 Materials

3,4-Ethylenedioxythiophene (EDOT, AR 99%), styrene (St), *n*-butyl acrylate (BA, 99+%), potassium persulfate (KPS, 99+%), dimethyl sulfoxide (DMSO, ≥99%) and methanol (≥99%) were purchased from Aladdin.Sodium styrenesulfonate (SSNa, AR 99%) and iron(iii) ferric sulfate (Fe_2_(SO_4_)_3_, AR 99%) were purchased from Macklin. Reduced graphene oxide was purchased from Suzhou Tanfeng Graphene Tech Co., Ltd.

### 2.2 Synthesis of P(SSNa-BA-St) latex *via* emulsion polymerization

A total of 3 g SSNa, 4.9 g BA and 2.1 g St were mixed with 78 g water at 78 °C for 15 min under N_2_ with stirring in a round bottom flask, followed by addition of the 0.1 g KPS through a constant flow pump. After stirring at 78 °C for 4 h, the emulsion polymerization was finished. The NPs were washed twice by centrifugation at 10 000 rpm for 30 min and ready to use.

### 2.3 Synthesis of P(SSNa-BA-St)/PEDOT elastic conductive dispersions

15 g of the prepared P(SSNa-Ba-St) emulsion and 0, 1, 2, 4, 5 g EDOT (0.3, 0.7, 1.1, 1.5 wt%) were separately mixed with 15 g water followed by 15 min sonication. 0.005 g ferric sulfate in 10 g water and 2.85 g KPS in 60 g water were separately added into two constant pressure dropping funnels. Extra water was added into the flask to keep the total mass of the content as 135 g. Then the two mixture in funnels were dropped into the flask for 2 h at the same time under N_2_, followed by stirring overnight at 30 °C. Then the P(SSNa-Ba-St)/PEDOT elastic conductive dispersions was prepared.

### 2.4 Synthesis of P(SSNa-BA-St)/PEDOT/graphene elastic conductive dispersions

15 g of the prepared P(SSNa-BA-St) emulsion and 0, 1, 2, 4, 5 g EDOT (0.3, 0.7, 1.1, 1.5 wt%) were mixed with 15 g water followed by 30 min sonication. Then 0, 1, 2, 4, 5 g graphene (7.4 × 10^−4^, 1.5 × 10^−3^, 3.0 × 10^−3^, 3.7 × 10^−3^ wt%) was added into the flask with 30 min sonication and 1 h stirring at 30 °C under N_2_. 0.005 g sulphate heptahydrate in 10 g water and 2.85 g KPS in 60 g water were separately added into two constant pressure dropping funnels. Extra water was added in to the flask to keep the total mass of the content as 135 g. Then the two mixture were dropped into the flask for 2 h at the same time under N_2_ and stirring followed by stirring overnight at 30 °C. A flow of N_2_ was used to prevent the overoxidation of PEDOT which might lead to conductivity deceasing. Then the P(SSNa-Ba-St)/PEDOT elastic conductive dispersions was prepared.

### 2.5 Preparation of elastic conductive thin films

An appropriate amount of doping agent, DMSO and methanol could be blended with the purified P(SSNa-Ba-St)/PEDOT or P(SSNa-Ba-St)/PEDOT/graphene dispersion in advances.The mixed solution was dropped cast on a glass plate and then dried at 50 °C for 3 h to form the P(SSNa-Ba-St)/PEDOT or P(SSNa-Ba-St)/PEDOT/graphene elastic conductive composite film.

### 2.6 Characterization

To determine the hydrodynamic diameter and zeta potential of the P(SSNa-Ba-St) latexes, dynamic light scattering (DLS) and zeta potential measurements were carried out using a Zetasizer Nano ZS system (Malvern Instruments, Ltd., UK). DTS Application 5.10 software was employed to analyse the data obtained. The chemical groups of the nanoparticles were analysed by Fourier transform infrared (FTIR) (Thermo NICOLET iS10) in transmission mode. For that, dried material powders were mixed with KBr (40 mg) and then formed into a disc in a manual press. Transmission spectra were recorded using at least 32 scans with 4 cm^−1^ resolution in the spectral range 4000–400 cm^−1^. The glass transition temperature (*T*_g_) of each sample was determined using a differential scanning calorimeter (DSC) (Model DSC 3, Mettler-Toledo International Inc., Columbus, OH). 12 mg of dispersions dried powders were sealed into a 40 μL aluminium pan. An empty pan was used as a reference sample. The temperature scan for each sample was measured from −50 °C to 50 °C at a heating rate of 5 °C per minute. The data acquired by the DSC were analysed by STAR Thermal Analysis software (NETZSCH 200F3) to determine the onset and endset glass transition temperatures.

The surface resistance was measured using an ohmmeter. The conductivity of the composite films was evaluated by the following equation: 
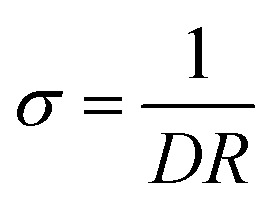
, where *R* represents the surface resistance (Ω sq^−1^), *D* is the film thickness (cm). Equal width and length were used throughout this study. The optical transmittance spectra of the conductive composite films in the visible light wavelength range (300–700 nm) were detected using a UV-visible spectrometer. The contact angle was measured by Powereach JC 2000D.

Bending tests of the PSS/PEDOT and P(SSNa-Ba-St)/PEDOT/graphene film were performed to detect the flexibility. The conductive dispersions were coated on flexible poly(ethylene terephthalate) substrates to form the specimens. The bending test was executed by bending the specimens back and forth (180°) 100 times.

## 3. Results and discussion

**Scheme 1:**
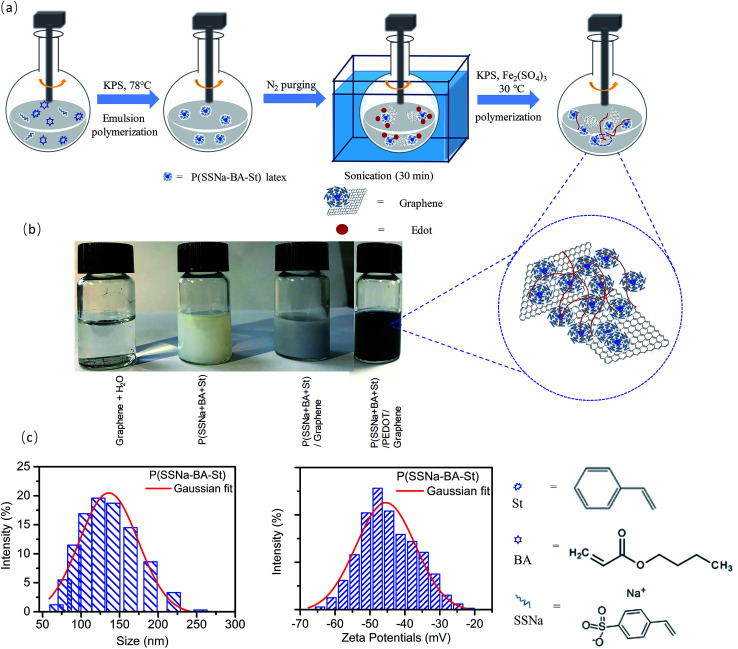
(a) Synthesis process of P(SSNa-BA-St)/PEDOT/graphene dispersions. (b) Photographs of dispersion of graphene in water, P(SSNa-BA-St) latex emulsions, P(SSNa-BA-St)/graphene and P(SSNa-BA-St)/PEDOT/graphene dispersions. (c) Hydrodynamic diameters and zeta potentials of P(SSNa-BA-St) latex.

The P(SSNa-BA-St)/PEDOT/graphene were synthesized as shown in Scheme 1(a). SSNa was used as emulsifier to stabilize the polymerization and also a monomer to mixed with two other elastic monomers BA and St. The copolymerized latexes were achieved by emulsion polymerization under the initiation by KPS. As shown in Scheme 1(c), the average hydrodynamic diameter of the P(SSNa-BA-St) latex was *ca.* 128 nm by intensity (PDI: 0.08) and the average zeta potentials was *ca.* −50 mV. With the help of efficient stabilizer SSNa, the size of the latex was in nanometre range with low PDI which provide large surface area to polymerize EDOT on the nanoparticles. And the strong negative surface charges make them serve as good charge compensator of positively charged PEDOT and also provide the high colloidal stability of the nanoparticles. Then the P(SSNa-BA-St) latex were applied as a template for both EDOT polymerization and graphene–water dispersion. As shown in Scheme 1(b), the redox graphene oxide cannot disperse into water, however, they can disperse into P(SSNa-Ba-St) emulsion well without aggregation and then the P(SSNa-Ba-St)/graphene can be used as template to polymerize EDOT under KPS/ferric sulfate oxidative. No further reduction processes by applying toxic chemicals or high temperature were needed. These dispersions are stable for more than 6 months. The graphene locating on the surface of P(SSNa-BA-St)/PEDOT particles are due to the hydrogen bonding and stacking interaction (strong π–π interaction with aromatic structure) between not only PSSNa and graphene but also PSt and graphene, which provide very stable water dispersions.^13,23^

**Fig. 1:**
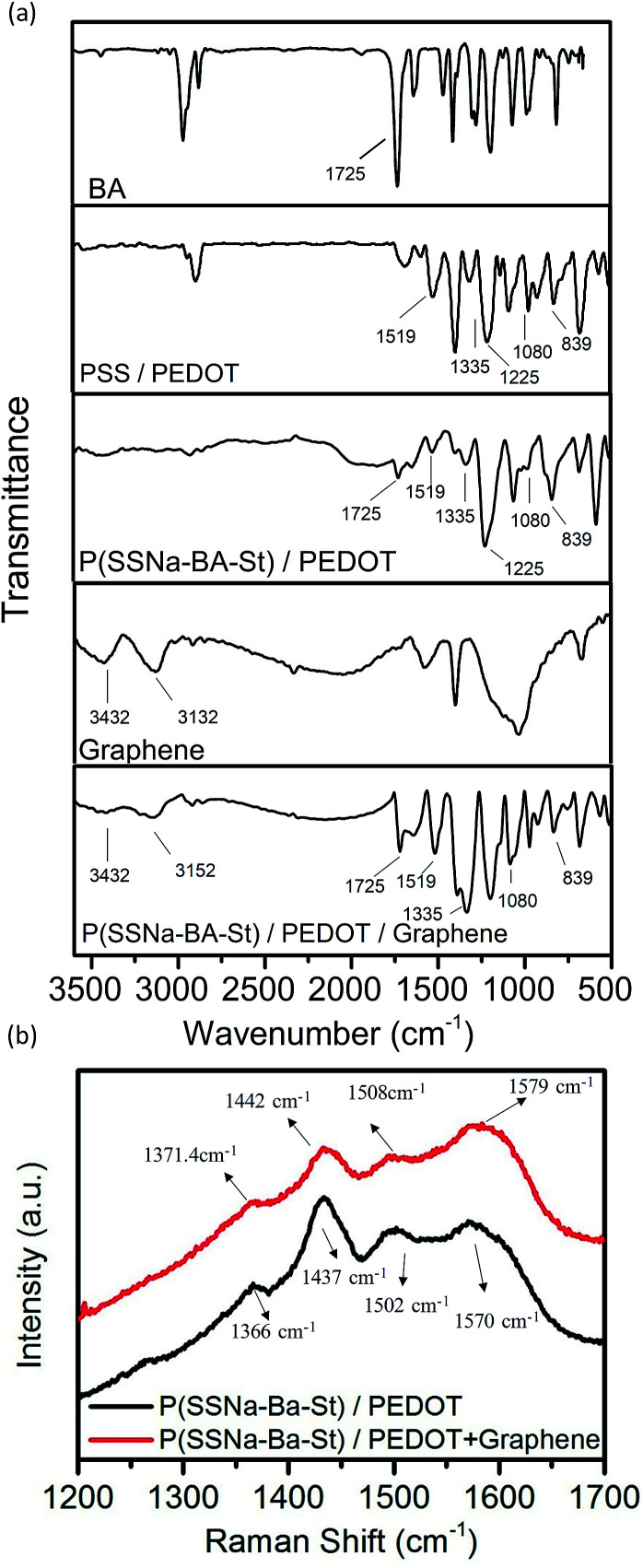
(a) FTIR spectra and (b) Raman spectra of P(SSNa-BA-St)/PEDOT and P(SSNa-BA-St)/PEDOT/graphene.

In order to investigate the components of the conductive film, the typical FTIR spectrum of the extracted composite polymer was shown in Fig. 1(a). For all of PSS/PEDOT, P(SSNa-BA-St)/PEDOT and P(SSNa-BA-St)/PEDOT/graphene composite, the vibrations at 839 cm^−1^, 1080 cm^−1^, 1335 cm^−1^, and 1519 cm^−1^ are corresponding to the C–S bond in thiophene rings; –SO_2_, and –SO_3_^−^ in PSS; C–C stretching in thiophene rings and C=C asymmetrical stretching in thiophene rings, respectively, indicating the successful incorporating the PSS in the system.^24^ The peaks at 1725 cm^−1^ of PEDOT/P(SSNa-BA-St) and PEDOT/P(SSNa-BA-St)/graphene was associated with C=O stretch of BA.^25^ The extra peaks at 3152 cm^−1^ and 3432 cm^−1^ for PEDOT/P(SSNa-BA-St)/graphene are attributed to the remaining –OH group on the surface of the graphene, which are typical peaks for reduced graphene oxide, and it confirms the formation of graphene dispersed PEDOT/P(SSNa-BA-St) dispersions.^23,26^

The protection effect of PSS and electron-stacking interaction between graphene and P(SSNa-BA-St)/PEDOT are confirmed by Raman spectroscopy. The Raman spectrum of graphene applied in this work was consistent of the typical reduced graphene oxide spectrum as shown in Fig. S1.† The G-band which is very sensitive to the number of layers of the graphene was shown at 1584 cm^−1^, owing to the first-order scattering of the E_2g_ phonons and the D band exhibited at 1343 cm^−1^ which is attributed to the breathing mode of *κ*-point phonons of A_1g_ symmetry.^27^ Based on the empirical equation, *ω*_G_ = 1581.6 + 11/(1 + *n*^1.6^),^28^ where *ω*_G_ is the band position in wavenumbers and *n* is the number of layers present in the sample, the number of layers of the graphene can be estimated as two. And also, the 2D band in Fig. S1† which can be fitted by four Lorentz peaks was consistent with the bilayer graphene spectrum in the literature.^28,29^ However, the D band exhibited very high intensity (*I*_D_/*I*_G_ = 1.1) indicating the disorder or the defect present in the graphene.^29,30^ For the P(SSNa-BA-St)/PEDOT in Fig. 1(b), the four typical bands were attributed as a C=C anti-symmetric stretching (1570 cm^−1^), C=C asymmetrical stretching (1502 cm^−1^), C=C symmetrical stretching (1437 cm^−1^) and single C–C stretching (1366 cm^−1^).^31^ Comparing with the PEDOT/P(SSNa-BA-St), the graphene doped PEDOT/P(SSNa-BA-St) exhibited slightly shifted (for example, from 1437 cm^−1^ to 1442 cm^−1^ and from 1570 cm^−1^ to 1579 cm^−1^) according to the strong π–π interaction of aromatic structures of PEDOT/P(SSNa-BA-St) and electron-rich graphene, illustrating the successful doping of graphene into PEDOT/P(SSNa-BA-St).^23,32^

**Fig. 2:**
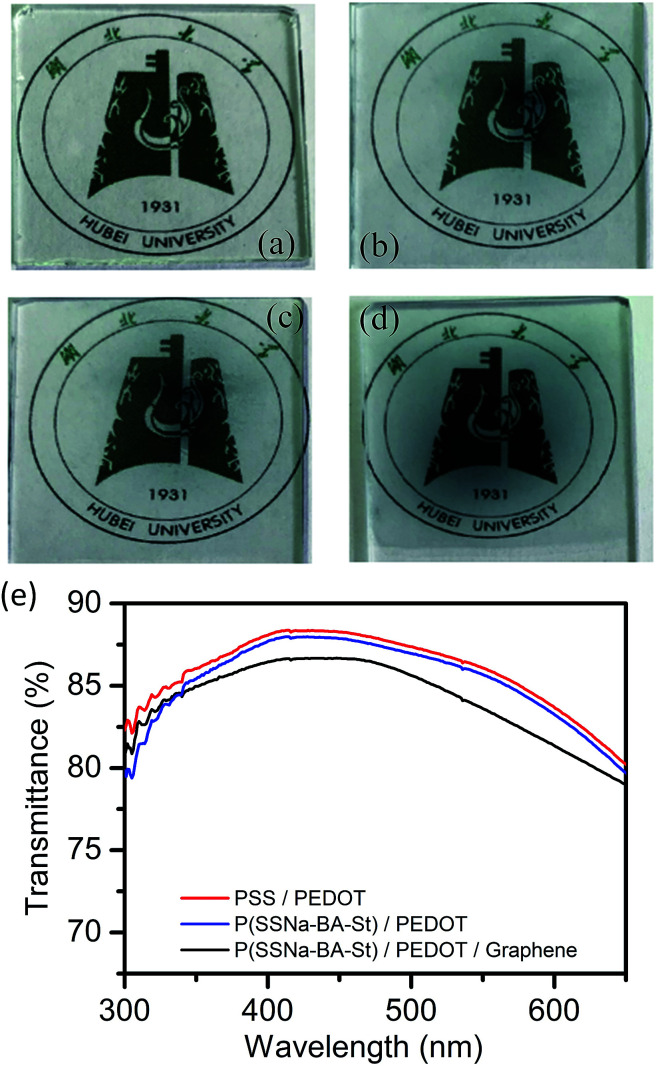
(a) Photographs of glass, (b) PSS/PEDOT, (c) P(SSNa-BA-St)/PEDOT and (d) P(SSNa-BA-St)/PEDOT/graphene film coated on a glass substrate. (e) Transmittance spectra of PSS/PEDOT, P(SSNa-BA-St)/PEDOT and P(SSNa-BA-St)/PEDOT/graphene composite films.

The conductive dispersions were drop casting on glass coverslips and PSS/PEDOT (Fig. 2(b)), P(SSNa-BA-St)/PEDOT (Fig. 2(c)) and P(SSNa-BA-St)/PEDOT/graphene (Fig. 2(d)) films all exhibit a good coating property without aggregation on the glass with good transparency. The transmittances of all three films (Fig. 2(e)) were measured as 83–86% at 550 nm which indicates that the P(SSNa-BA-St)/PEDOT and graphene doped P(SSNa-BA-St)/PEDOT films exhibit the similar relatively good transparency with PSS/PEDOT.

**Fig. 3:**
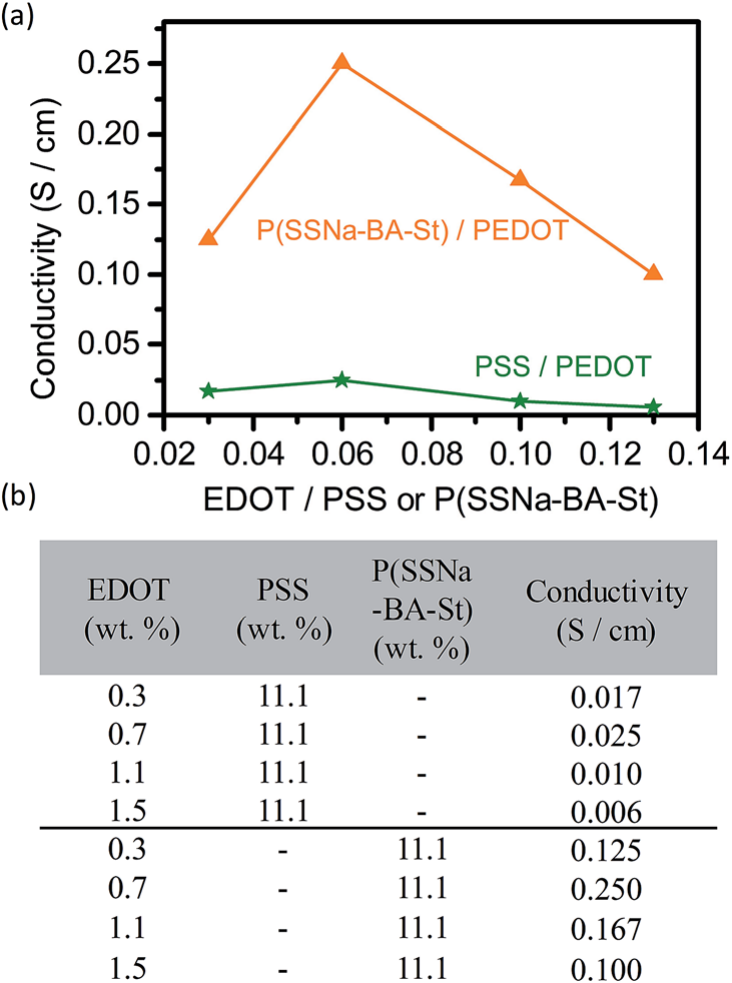
(a) Conductivity of PSS/PEDOT and P(SSNa-BA-St)/PEDOT with the concentration ratio of EDOT increasing. (b) Relative weight ratio of three components and conductivities.

In order to investigate the optoelectronic properties, P(SSNa-BA-St)/PEDOT were drop casting on the glass substrate to form the well mixed uniform conductive films. The conductivity of PSS/PEDOT and P(SSNa-BA-St)/PEDOT films were shown in Fig. 3(a) and (b). With the content ratio of EDOT to PSS increasing from 0.03 to 0.06, the conductivities increase from 0.017 S cm^−1^ to 0.025 S cm^−1^ owing to the concentrations of conducive component increasing, followed by the decreasing to 0.006 S cm^−1^ owing to the precipitation formation of excessive PEDOT, indicating the best doping concentration of EDOT to PSS here was 0.06. More important, instead of PSS, the conductivity trend of applying P(SSNa-BA-St) as the template for PEDOT polymerization follows the same with PEDOT/PSS but exhibit much higher conductivities than them. The sample of EDOT to PSS(SSNa-BA-St) ratio as 0.06 provides the highest conductivity as 0.25 S cm^−1^ which is 10 times higher than PEDOT/PSS with the same content ratio. The improvement of conductivity is owing to the small nanoparticles sizes (approximately 128 nm) of P(SSNa-BA-St) latex providing larger surface area to polymerize the extended PEDOT chain on the surface of the nanoparticles, leading to higher conductivity than PSS/PEDOT that having grains with hydrophobic coiled PEDOT chains in the core and hydrophilic insulator PSS as the shell.

By controlling the EDOT to PSS ratio as 0.06, the conductivity of the graphene doped P(SSNa-BA-St)/PEDOT were shown in Table 1. With the graphene to EDOT ratio increasing from 0 to 0.004, the conductivities of P(SSNa-BA-St)/PEDOT/graphene films increase from 0.250 S cm^−1^ to 2.500 S cm^−1^ which is higher than the reported PEDOT/graphene composite layers (0.13–0.20 S cm^−1^).^33,34^ The 10 times conductivity increasing compared with P(SSNa-BA-St)/PEDOT and 100 times increasing comparing with PSS/PEDOT are due to the help of unique electrical properties of graphene and also indicates that the graphene were successfully doped on the surface of P(SSNa-BA-St)/PEDOT particles through hydrogen bonding and strong π–π interaction of graphene with PSS and PSt. Then the conductivity dropped down to 0.625 S cm^−1^ by reaching the graphene to EDOT ratio at 0.005, owing to the excess of graphene precipitated out of the solution. In addition, the conductivity of P(SSNa-BA-St)/PEDOT/graphene films can also be enhanced by applying the solvent effect. As shown in Table 1, the conductivity of DMSO and methanol doped or post-treated P(SSNa-BA-St)/PEDOT/graphene (graphene to PEDOT ratio as 0.004) films exhibit almost 2.2 times higher than the relative untreated films and 220 times higher than the untreated PSS/PEDOT film, which might be due to the phase separation between the conducting PEDOT chains and insulating PSS chains.

**Table 1:** Conductivities of films with different graphene to PEDOT ratios under different film preparation methods

Graphene:PEDOT	Conductivity (drop casting) (S cm^−1^)	Conductivity (mix with DMSO 20 h, drop casting) (S cm^−1^)	Conductivity (drop casting, DMSO) (S cm^−1^)	Conductivity (drop casting, methanol) (S cm^−1^)
0	0.250	0.500	0.500	0.500
0.001	0.625	1.000	1.250	0.833
0.002	0.833	1.250	1.667	1.250
0.004	2.500	5.556	5.556	5.000
0.005	0.625	0.833	0.714	0.714

**Fig. 4:**
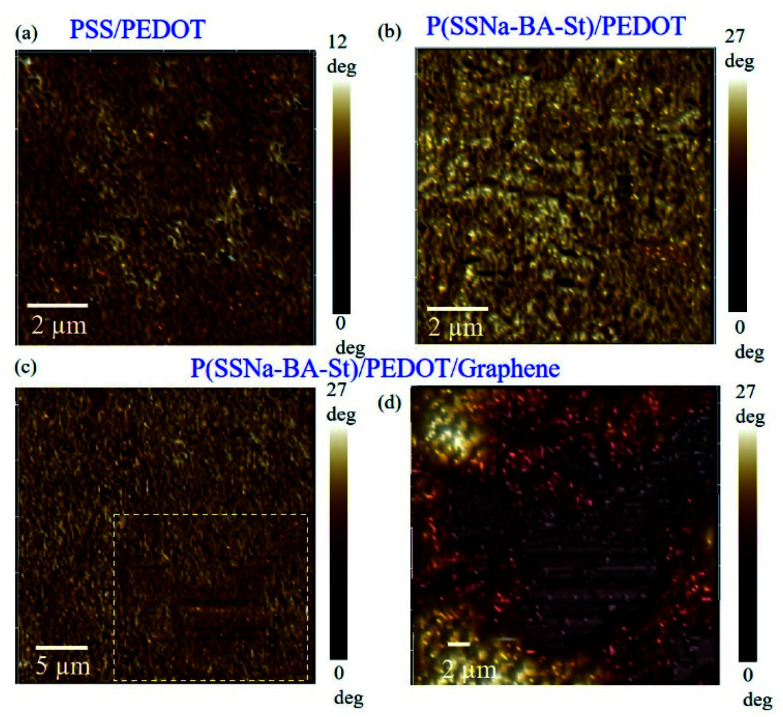
Corresponding phase images of (a) PSS/PEDOT, (b) P(SSNa-BA-St)/PEDOT, (c) P(SSNa-BA-St)/PEDOT/graphene films and (d) zoom in of (c) of the topography images obtained with tapping-mode AFM.

Atomic force microscopy (AFM) was applied to reflect the morphological changes in the films. Fig. 4(a) shows the phase AFM image of PSS/PEDOT on tapping mode and small grains were observed on the surface, which is attributed to the PEDOT-rich grains surrounded by thin PSS layers. The bright regions corresponds to PEDOT-rich areas and the dark regions corresponds to PSS-rich areas.^35^ However, the P(SSNa-BA-St)/PEDOT films exhibited elongated grains and form longer connected networks of PEDOT-rich area and the phase segregate of P(SSNa-BA-St) and PEDOT was shown in Fig. 4(b).^36^ This phenomenon demonstrated that the conductivity improvement from PSS/PEDOT to P(SSNa-BA-St)/PEDOT is also attributed to an increase of phase separation. The graphene sheet of P(SSNa-BA-St)/PEDOT/graphene film was observed by AFM images which illustrated the graphene sheet was stabilized in the system and located in the P(SSNa-BA-St)/PEDOT matrix, as shown in Fig. 4(c) and (d).

**Fig. 5:**
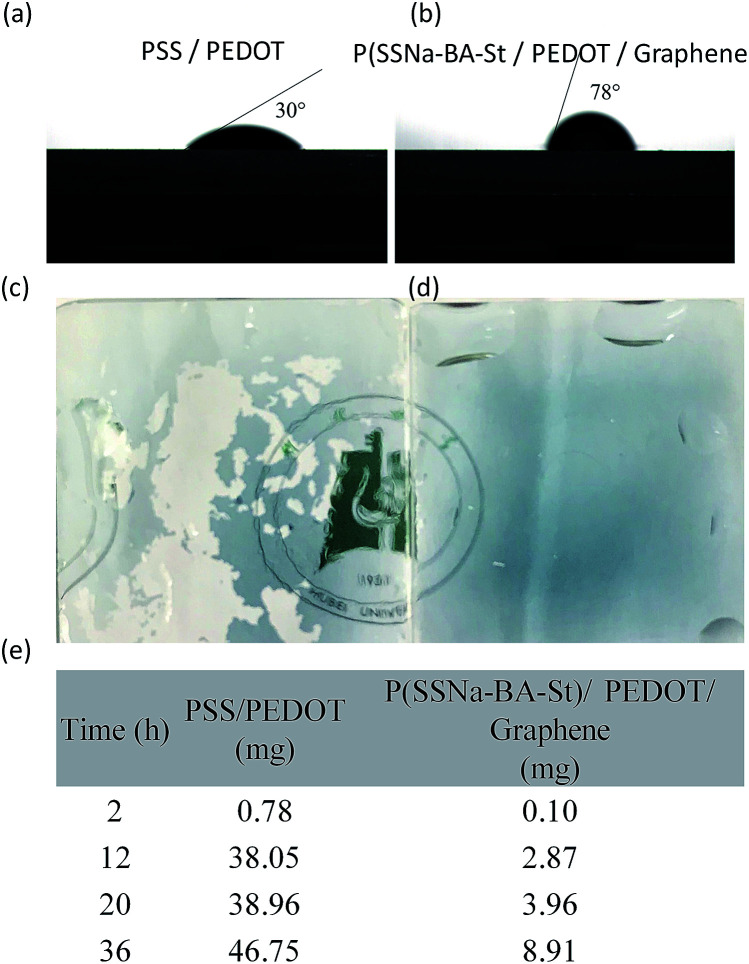
The photograph of water drops contacts angle tests of (a) PSS/PEDOT and (b) P(SSNa-BA-St)/PEDOT/graphene composite films. The photograph of the (c) PSS/PEDOT and (d) P(SSNa-BA-St)/PEDOT/graphene films after water resistance test. (e) The moisture absorption of PSS/PEDOT and P(SSNa-BA-St)/PEDOT/graphene films in air with different time.

In addition, the hydrophobicity of as prepared films was also investigated. The high hydrophilic PEDOT:PSS film exhibit the water contact angle at 30°, however, the contact angle of P(SSNa-BA-St)/PEDOT/graphene film can reach to 78° which is much higher than the reported 4.8–7.0° for PSS/PEDOT/graphene,^37^ indicating their excellent waterproof ability due to the copolymerization of water insoluble monomers BA and St, as shown in Fig. 5(a) and (b). Furthermore, the water resistance test was also performed. The PSS/PEDOT and P(SSNa-BA-St)/PEDOT/graphene films were swollen in water with a water flow. As shown in Fig. 5(c), the PSS/PEDOT would swell in the water firstly, and then the large portion was broken into pieces in the water flow. On the contrary, the P(SSNa-BA-St)/PEDOT/graphene film could remain the original shape on the substrate, indicating their excellent water resistance ability (Fig. 5(d)). As shown in Fig. 5(e), the moisture absorption of PSS/PEDOT film in air increase dramatically with time, however, the P(SSNa-BA-St)/PEDOT/graphene film shows relatively much smaller amount of moisture absorption. After leaving in air for 12 h, the moisture absorption of PSS/PEDOT film is 13.3 times higher than P(SSNa-BA-St)/PEDOT/graphene films, which is also due to the copolymerization of water proof BA and St monomers.

**Fig. 6:**
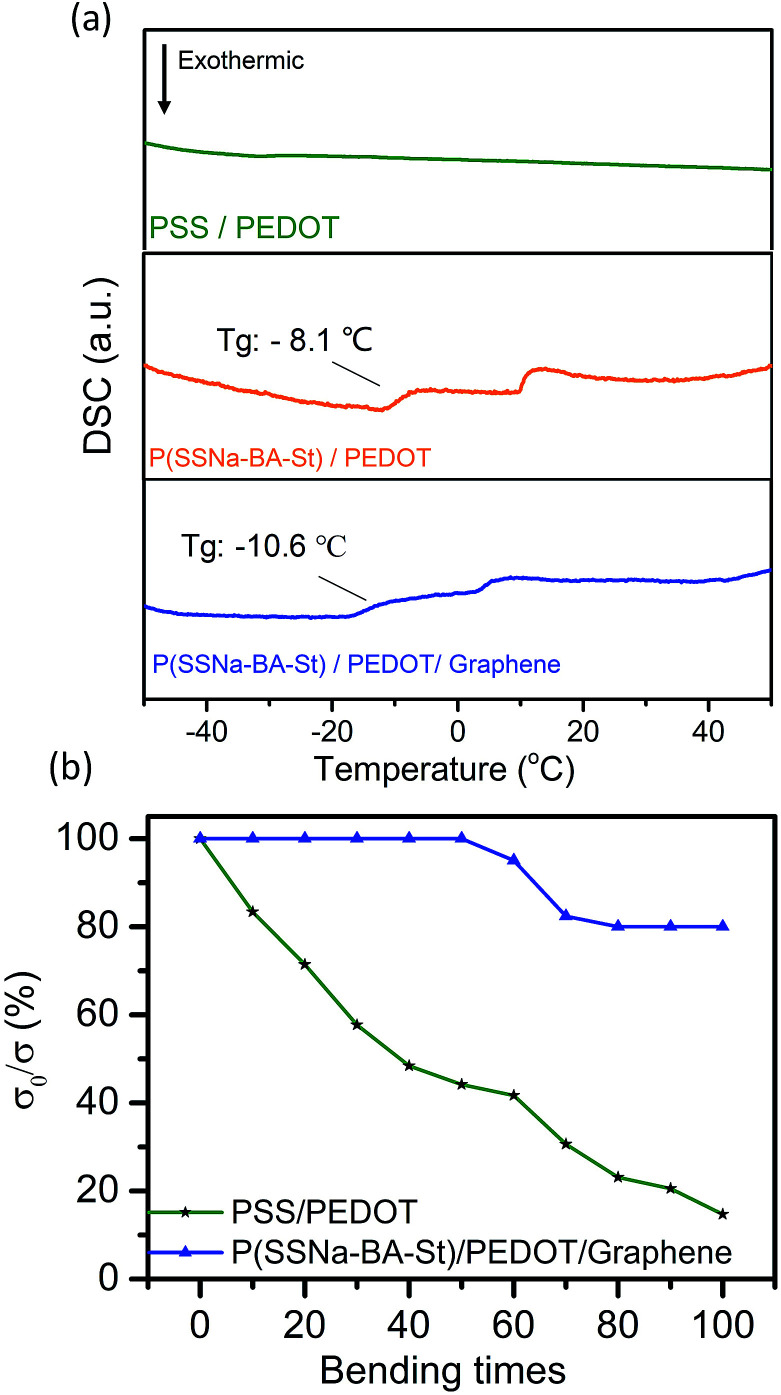
(a) DSC results of the PSS/PEDOT, P(SSNa-BA-St)/PEDOT and P(SSNa-BA-St)/PEDOT/graphene. (b) The relative surface resistance ratio of the PSS/PEDOT and P(SSNa-BA-St)/PEDOT/graphene films with different bending times.

Fig. 6(a) shows the differential scanning calorimetry (DSC) thermograms of the three types of polymers. PSS/PEDOT does not have a well-defined *T*_g_ but the DSC results showed that the *T*_g_ for P(SSNa-BA-St)/PEDOT was around −8.1 °C and P(SSNa-BA-St)/PEDOT/graphene was around −10.6 °C which can be attributed to the introduction of the lower *T*_g_ material BA and St. The decreases of *T*_g_ could provide the excellent flexibility and elasticity to the films. The bending tests were also performed in Fig. 6(b). PSS/PEDOT and P(SSNa-BA-St)/PEDOT/graphene were coated on PET substrate and the flexibility of the continuous conductive film was determined. To avoid the influence of the initial surface resistance on the result of the bending test, the surface conductivity ratio (*σ*_0_/*σ*) was used, where *σ*_0_ and *σ* were the surface conductivity before and after bending, respectively. For the pure PSS/PEDOT specimen, the surface conductivity decreases to 15% of the original after 100 times bending cycles while P(SSNa-BA-St)/PEDOT/graphene remain 83% of the original. This indicated that the introduction of P(SSNa-BA-St) latex could strongly enhance the flexibility of conductive films compared with the conventional PSS/PEDOT film.

## 4. Conclusions

We reported an innovative transparent, elastic and flexible conductive composite materials P(SSNa-Ba-St)/PEDOT/graphene which were prepared by using P(SSNa-Ba-St) latex as template for both graphene and PEDOT. No extra surfactant is needed and monodispersed nanoparticles are formed. The conductivity of P(SSNa-BA-St)/PEDOT/graphene exhibit 220 times higher than the untreated PSS/PEDOT, which are due to the phase separation between the conducting PEDOT chains and insulating P(SSNa-Ba-St) chains. And P(SSNa-BA-St) latex have large surface area to polymerize the extended PEDOT chain on the surface of the nanoparticles, which provide higher conductivity than PSS/PEDOT that having grains with hydrophobic coiled PEDOT chains in the core and hydrophilic insulating PSS as the shell. Furthermore, the water contact angle of P(SSNa-BA-St)/PEDOT/graphene film can reach to 78° and remain the original shape on the substrate under water flow, and also, the moisture absorption of P(SSNa-BA-St)/PEDOT/graphene films is 13.3 times lower than of PSS/PEDOT film after leaving in air for 12 h, which indicates an excellent water resistance ability due to the copolymerization of water proof BA and St monomers. The relatively low *T*_g_ of (SSNa-BA-St)/PEDOT/graphene film provide the excellent flexibility and elasticity to the films. Therefore, the P(SSNa-Ba-St)/PEDOT/graphene presented here exhibited transparent, good water resistance, low moisture adsorption, highly elastic and highly conductive properties, which can serve as a practical approach to fabricate the flexible, conductive and transparent films for wearable and implantable electronic devices, and photovoltaic cells.

## Conflicts of interest

There are no conflicts to declare.

## Supplementary Material

RA-009-C9RA09099A-s001
