# Supplementary material for: Highly elastic and flexible transparent conductive films derived from latex copolymerization: P(SSNa-BA-St)/PEDOT/graphene
Source: RSC Adv. 2019 Dec 20;9(72):42335–42. doi: 10.1039/c9ra09099a (PMC9076566; doi:10.1039/c9ra09099a)
Supplement: RA-009-C9RA09099A-s001 [file RA-009-C9RA09099A-s001.pdf]

Electronic Supplementary Material (ESI) for RSC Advances.
This journal is © The Royal Society of Chemistry 2019

Supplementary Information

Highly elastic and flexible transparent conductive films derived from latexes copolymerization: P(SSNa-BA-St)/PEDOT/Graphene

Bo Huang,[a] Xinxin Luo,[a] Qichao Zou,[a] Suxiao Wang,[a] and Jinzhi Zhang*[a]*

[a] Ministry of Education Key Laboratory for the Synthesis and Application of Organic Functional Molecules, Hubei Collaborative Innovation Centre for Advanced Organic Chemical Materials, College of Chemistry and Chemical Engineering, Hubei University, Wuhan, 430062, China.

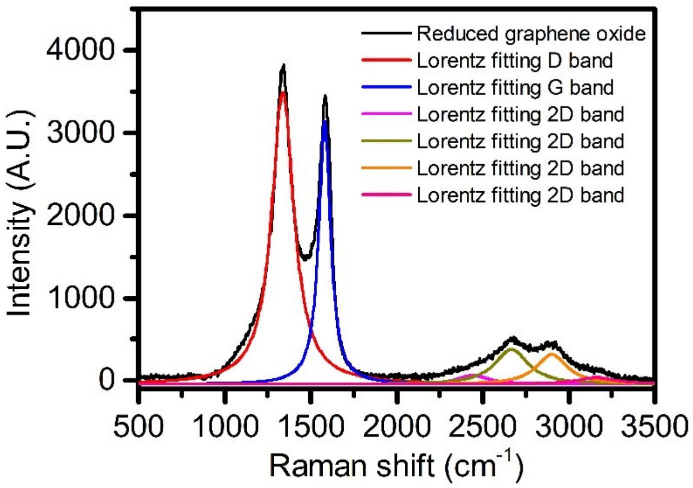

Figure S1. Raman spectrum of the pure Graphene.
